# Derbyshire Royal Infirmary

**Published:** 1895-02-16

**Authors:** 


### HOSPITAL ADMINISTRATION.

[Questions and correspondence, marked "Hospital Administration" on the envelope, addressed to the Editor, are invited for this column.]

### DERBYSHIRE ROYAL INFIRMARY.

A correspondent writes with reference to our remarks on page 335 of The Hospital, that the extra physician and extra surgeon will not only have no beds, but "they are the underlings of the present medical staff. As a consequence, the only F.R.C.S. in the town declines the new office, although he is a gentleman in the prime of life, and in active practice of the best kind, and of high reputation." Our correspondent, however, describes himself as "quite hopeless," and proceeds, "There seems no help but the reduction of this fine institution to the level of a workhouse infirmary," owing to the "fact of the qualifications being so low." We much regret to find that we were misled as to the duties of the new members of the honorary medical staff, and hope that the committee may see their way to reconsider Rule 43, so as to secure that each physician and surgeon shall have a certain number of beds alloted to him, and that the out-patient department work, which is much more important than our correspondent seems to realise, shall be fairly apportioned between the members of the staff. As we said before, it would be a grievous calamity, having regard to the large sums of money spent upon the new buildings, and the opportunities offered for the highest kind of clinical and pathological work, if the Royal Infirmary, Derby, should be allowed to fail, by becoming for the most part a mere resting place for chronic cases, where little or no attempt was made to keep the medical work up to the highest standard of modern hospital requirements and knowledge.

